# Characteristics of the four subtypes of myelodysplastic/myeloproliferative neoplasms

**DOI:** 10.3892/etm.2013.975

**Published:** 2013-02-26

**Authors:** HUANLING WU, SHUQUAN BIAN, JINGXUE CHU, XIAOYAN ZHONG, HUI SUN, BINGCHANG ZHANG, ZHIMING LU

**Affiliations:** 1 Clinical Laboratory of Provincial Hospital Affiliated to Shandong University, Jinan, Shandong 250021;; 2 Clinical Laboratory of Xintai Hospital Affiliated to Taishan Medical University, Jinan, Shandong 271200;; 3 Clinical Laboratory of Jinan Central Hospital, Jinan, Shandong 250013;; 4 Clinical Laboratory of the Eighth Hospital in Jinan, Jinan, Shandong 250014;; 5 Shandong Center For Clinical Laboratories, Jinan, Shandong 250021, P.R. China

**Keywords:** myelodysplastic, myeloproliferative, chronic, neoplasms, dysplasia, immunology, cytogenetic, molecular genetic

## Abstract

The aim of the present study was to investigate the characteristics of the four subtypes of myelodysplastic/myeloproliferative neoplasms (MDS/MPNs) in order to improve current knowledge and to aid their diagnosis. A total of 53 cases of MDS/MPNs were analyzed using routine blood cell analysis and morphological, cytogenetic and molecular genetic characteristics were investigated. Numerical data for several groups were compared using a single-factor analysis of variance. The Student-Newman-Keuls test was used to compare the means of two groups. The proportions were compared using a Chi-square test or Fisher’s exact test. Analysis of the patients with MDS/MPNs revealed that 46 patients (86.8%) had paleness and fatigue, and blood analysis revealed hemoglobin (Hb) levels of 83.1±24.6 g/l, a white blood cell (WBC) count of 19.8±8.1×10^9^/l and a platelet (PLT) count of 158.7±108.2×10^12^/l. Immature neutrophils and monocytes were identified in the peripheral blood at levels of 0.058±0.031 and 0.152±0.034%, respectively. There were 23 cases (43.4%) with dyserythropoiesis and 36 cases (67.9%) had dysgranulopoiesis. Fifteen cases were immunologically characterized using flow cytometry (FCM), of which 13 cases showed abnormalities on blasts and myelocytes. Karyotyping was performed in 27 cases of MDS/MPN and 12 (44.4%) were identified as abnormal. In 23 cases, testing for BCR/ABL1, AML-ETO, CBF-MYH11A, PML-RARA, E2A-PBX1, TEL-AML1, SIL-TAL1 returned negative results. The JAK2V617F mutation was positive in one of five cases. The majority of MDS/MPN cases had anemia, cytosis, low-grade blasts and immature neutrophils in the peripheral blood and dysplasia in the bone marrow. Immunological abnormalities and abnormal karyotypes occurred frequently in MDS/MPNs and although there were no statistical differences between the four subtypes, these were able to aid diagnosis. No specific molecular abnormalities were identified in MDS/MPNs.

## Introduction

In 2001, the World Health Organization (WHO) revised the hematopoietic and lymphoid tissue tumor classification and defined a new type of myeloid tumor called a myelodysplastic/myeloproliferative neoplasm (MDS/MPN). It is a rare clonal myeloid neoplasm that exhibits overlapping myeloproliferative and myelodysplastic features at initial presentation. It exhibits a reduced peripheral blood cell count, which is caused by dysplasia and is characteristic of MDS, and excessive apoptosis and cytosis, which is caused by a series of one or more lines of cell proliferation and occurs in MPN. In 2008, WHO established the criteria for classifying hematopoietic and lymphoid neoplasms (1) and defined MDS/MPNs to include four major subcategories: Chronic myelomonocytic leukemia (CMML), atypical chronic myeloid leukemia (BCR-ABL1-negative; aCML), juvenile myelomonocytic leukemia (JMML) and MDS/MPN, unclassifiable (MDS/MPN-U), including refractory anemia with ring sideroblasts and thrombocytosis (RARS-T). CMML with eosinophilia and PDGFRB rearrangement were moved into a new genetically defined category.

Previous studies have focused on CMML, JMML or aCML alone. Although MDS/MPNs have been studied, their diagnosis remains difficult. In the present study, we analyzed the morphological, immunological*,* cytogenetic and molecular genetic features of MDS/MPNs, to improve understanding of and distinguish between MDS/MPN subtypes and to investigate their association with prognosis.

## Patients and methods

### Patients

A total of 53 patients, including 33 males and 20 females aged 45 days to 84 years old, were diagnosed or retrospectively diagnosed with MDS/MPN based on the clinical manifestations and morphological, immunological, cytogenetic and molecular biological analysis of the peripheral blood and bone marrow cells, in the Provincial Hospital Affiliated to Shandong University, between August 2002 and August 2010. The present study was conducted in accordance with the Declaration of Helsinki and with the approval of the Ethics Committee of Provincial Hospital Affiliated to Shandong University (Shandong, China). Written informed consent was obtained from all participants. The study included 24 patients with CMML, who had a median age of 57 years (33–71 years), 13 patients with aCML, who had a median age of 68 years (16–84 years), 12 patients with JMML, who had a median age of 4 years (45 days-16 years) and 4 patients with MDS/MPN-U, who had a median age of 68 years (57–84 years). None of the patients had MDS, MPN, cytotoxic drugs or cytokine therapy, which may have caused the myelodysplastic or myeloproliferative features.

### Routine blood analysis

Peripheral or intravenous blood was collected and mixed with 2 ml of EDTA-K_2_ anticoagulant. The blood was analyzed to determine hemoglobin (Hb), white blood cell (WBC), eosinophil (EC), basophil (BC) and platelet (PLT) levels, which were counted using a Sysmex XE-2100 automated hematology analyzer (Shandong Zhixin Medical Company, Jinan, China). Blood smears were stained using the Wright-Giemsa method; 200 WBCs were classified and counted and the ratio of monocytes was calculated and converted into the absolute monocyte value, with particular attention to the morphological changes in granulocytes (blasts/immature), mononuclear cells (blasts/immature), red blood cells and platelets.

### Bone marrow/blood morphology

Cell morphology was observed using a Wright’s stain on bone marrow/blood films. Pathological hematopoietic cells were observed as follows: 500 bone marrow cells or 200 peripheral blood WBCs were examined and samples with ≥5% pathological cells (or ≥10% for cell lines) were considered positive (2,3).

Bone marrow aspirate smears were examined and the differential counts for 500 nucleated cells were obtained. The following morphological parameters were documented: Dysgranulopoieis included nucleus-cytoplasm asynchrony, nuclear hypolobation (pseudo-Pelger-Huet), hypogranularity and binucleated granulocytes; dyserythropoiesis was defined by karyorrhexis, megaloblastoid changes and multinuclearity; dismegakaryopoiesis was evaluated in terms of hypolobulated micromegakaryocytes, non-lobulated nuclei in megakaryocytes of all sizes and multiple widely separated nuclei. For the erythroid and myeloid lineages, the presence of these dysplastic features were confirmed in least 5 of the 500 nucleated cells from each lineage. For megakaryopoiesis, the dysplastic features were confirmed in at least 2 of the 25 megakaryocytes which were observed.

### Staining and immunohistochemical staining

Bone marrow smears from the anemic patients were routinely stained for iron. Iron particles were observed in erythroblasts and the ratio of ring sideroblast was determined. According to the International MDS Morphology Workshop, ≥5 iron particles covering ≥1/3 of an erythroblast nucleus should be defined as a ring sideroblast. α-naphthol butyrate esterase (α-NBE) was used to stain the specimens in which mononuclear cells and granulocytes were difficult to identify. The ratio of mono-nuclear cells was determined. MPO and non-specific esterase staining was performed in cases where naïve monocytes and immature granulocytes (or pathological granulocytes) were difficult to identify (4). The staining kit was purchased from Zhuhai Beisuo Biotechnology Company (Zhuhai, China). For cases with pathologically small megakaryocytes that were difficult to identify, the bone marrow film was stained with anti-CD41, the number of megakaryocytes was determined and the specimens were examined for megakaryocytic dysplasia (5). The kit was purchased from Shanghai Sun Biotechnology Company (Shanghai, China).

### Cellular immunology

BD-Calibur flow cytometry (FCM) was used to analyze blasts and myelomonocytes in the peripheral blood and bone marrow. Using EDTA as an anticoagulant for bone marrow and peripheral blood, 20*μ*l of antibody was added into each tube, which contained cells at 1×10^6^/l according to the following antibody panels: CD10-FITC/CD14-PE/CD45-ECD/CD64-PC5, HLA-DR-FITC/CD33-PE/CD45-ECD/CD15-PC5, CD5-FITC/CD34-PE/CD45-ECD/CD117-PC5, CD16-FITC/CD13-PE/CD45-ECD/CD11b-PC5, and CD7-FITC/CD56-PE/CD45-ECD/CD2-PC5 and CD45/SSC gating (6). All specimens were subjected to four-color FCM on a BD-Calibur flow cytometer using commercially available reagents. The red blood cells in the specimens were initially lysed with buffered ammonium chloride [Beckman Coulter (BC), Hialeah, FL, USA] and the remaining cells were washed once with phosphate-buffered saline containing bovine serum albumin (PBS-BSA) and sodium azide (pH 7.4) and resuspended to the desired cell concentration in PBS-BSA or minimum essential medium (MEM). Following this, 100 *μ*l of the cell suspension (5×10^5^ cells to 1×10^6^ cells) was incubated with the appropriate amount of titrated antibodies for 15 min at room temperature in the dark, washed once with PBS-BSA-azide and resuspended in 0.1% paraformaldehyde. The instrument alignment, sensitivity and spectral compensation were verified daily using standards, calibrators, procedural controls and normal peripheral blood samples prior to the processing of patient samples. The normal fluorescence intensity and maturation patterns were determined from 10 marrow samples for lymphoma staging and used as references. For each patient sample, the lymphocyte population was used as the internal control to ensure the correct voltage and compensation were applied. The changes in fluorescence intensity and maturation patterns were compared with the established data collected from normal bone marrow samples. In all cases, a minimum of 50,000 cells were obtained from each tube and analyzed by gating on erythroblasts/blasts/lymphocytes, maturing myeloid cells and monocytes. The myeloblasts were initially determined using CD45/SS and were then confirmed and enumerated using CD34.

### Cytogenetic analysis

Bone marrow samples (5 ml) were cultured for 24 h or 48 h, in accordance with standard procedures, using G-band analysis. The samples were observed for a minimum of 20 metaphases and karyotyped. The chromosomal abnormalities were described according to the ISCN 2005 recommendations.

### Molecular genetic analysis

The BCR/ABL1, AML-ETO, CBF-MYH11A, PML-RARA (L, S and V type), E2A-PBX1, TEL-AML1, SIL-TAL1 and JAK2V617F mutations were determined using real-time quantitative PCR. The primers were synthesized by Shanghai Shenyou Engineering Services Co., Ltd. (Shanghai, China).

### Statistical analysis

All data were analyzed using the Statistical Package for the Social Sciences (SPSS) version 13.0 for Windows (SPSS Inc., Chicago, IL, USA). First, the experimental data were analyzed for normality. If the data followed a normal distribution, the data were shown as mean ± SD. A single-factor analysis of variance (ANOVA) was used to compare the sample means of three groups. A Q test was used for pairwise comparison. The rates were compared using a Chi-square test. One-way ANOVA with Bonferroni correction was used to compare continuous variables among the different groups, whereas a Chi-square test was applied for categorical variables. P<0.05 was considered to indicate a statistically significant difference.

## Results

### Clinical symptoms

Of the 53 MDS/MPN cases, 33 were males and 20 were females (F/M = 1.65), with ages ranging from 45 days to 84 years. Fatigue and pallor occured in 86.8% of the MDS/MPN cases, 62.3% had splenomegaly, 39.6% had fever and 37.7% had ostealgia and rashes. Only 24.5% had lymphoid enlargement and even fewer had bleeding and hepatomegaly (Table I).

### Blood routine analysis

Anemia was identified in 75.4% of the MDS/MPN patients, the majority of which was mild or moderate, and only 9.4% developed severe anemia. In ∼81.5% of patients, the WBC was ≥10×10^9^/l and only 3.8% of patients had reduced WBC counts. The median WBC count was 19.8±8.1×10^9^/l. The WBC count in JMML was significantly higher compared with the other groups (P<0.05). The PLT count was decreased in 39.4%, normal in 22.6% and increased in 22.6% of patients. The mean PLT count was 158.7±108.2×10^9^/l, with the JMML group exhibiting the lowest count and the MDS/MPN-U group exhibiting the highest count. The differences in PLT count were significant between the JMML or MDS/MPN-U and the remaining two groups (P<0.05). The median Hb level in MDS/MPNs was 83.1±24.6 g/l and no significant differences were observed between the groups (P>0.05; Table II).

### Peripheral blood cell classification

All types of MDS/MPNs may result in blasts in the peripheral blood. The median blast percentage was 0.024±0.015% and the blast percentage for the CMML and aCML groups were significantly higher compared with the other two groups (P<0.05). MDS/MPNs also have different numbers of immature granulocytes in the peripheral blood, with a mean of 0.058±0.031%. aCML had the highest percentage compared with the other groups and this difference was significant (P<0.01). A pairwise comparison of the remaining three groups revealed no statistically significant differences (P>0.05). The mean monocyte ratio in MDS/MPNs was 0.152±0.034% and the ratio of mononuclear cells was increased most significantly in CMML, followed by JMML, however, this did not occur in aCML, MDS/MPN-U and other types of MDS/MPNs. The difference in monocyte ratio was significant between the three groups (P<0.05). The mean absolute monocyte value in the MDS/MPNs was 3.00±0.51×10^9^/l. The values in CMML and JMML increased the most significantly and the difference was significant compared with the two remaining groups (P<0.001). The mean peripheral blood absolute eosinophil value in the MDS/MPNs was 0.30±0.17×10^9^/l. The peripheral blood absolute eosinophil value of JMML was significantly higher than in the three other groups (P<0.05). The eosinophil count in the other subtypes was within normal limits. The mean peripheral blood absolute basophil value in the MDS/MPNs was 0.07±0.02×10^9^/l. The absolute basophil value was highest in aCML, however, this was not significantly different from the remaining three groups (P>0.05). The mean bone marrow blast ratio was 0.024±0.021% and the differences between CMML and the remaining three groups were significant (P<0.05; Table II).

### Analysis of immunology

FCM was performed on 15 cases and 13 were revealed to be abnormal. The most frequent abnormalities were blast percentages of ≥3% or blasts expressing lymphocytic antigens, including CD2/CD5/CD7. For granulocytes, the abnormalities were lower side scatter (SC) and asynchronous expression of CD14/CD64, CD13/CD15 and CD11b/CD13. In monocytes, the asynchronous expression of CD13/CD11b, CD15 and CD56 was identified (Table III).

### Bone marrow morphology analysis

Erythroid dysplasia was observed in 43.3% of the MDS/MPNs. The frequency was significantly higher in the MDS/MPN-U group compared with the three other groups (P<0.05). Myeloid dysplasia was observed in 67.9% of the MDS/MPNs. The frequency was highest in the aCML group, however, there were no significant differences between the four groups (P>0.05). Monocytic dysplasia was observed in 45.3% of the MDS/MPNs; the frequency was highest in the JMML group and the frequency in the MDS/MPN-U group was significantly lower than the three other groups (P<0.05). Megakaryocyte dysplasia was observed in 45.3% of the MDS/MPNs and this was most frequent in the MDS/MPN-U group, followed by the JMML and aCML group. There was no significant difference in the frequency of megakaryocyte dysplasia between these three groups, however they were significantly different from the CMML group (P<0.05; Table IV).

### Cytogenetic and molecular genetic analysis

Of the 53 MDS/MPNs cases, 27 were subjected to chromosome analysis and chromosome abnormalities were observed in 12 cases. The abnormalities are shown in Table IV. In the 12 MDS/MPN cases with chromosomal abnormalities, 3 cases (25%) of +8 and 4 cases (33.3%) of -7/7q^−^ were identified and these occured significantly more often than other chromosomal abnormalities (P<0.05). The chromosomal analysis of 27 MDS/MPN cases showed that the Ph- and AML-associated chromosomal abnormalities were negative and neither were independent of 5q- and inv(3)(q21q26). BCR/ABL, AML-ETO, CBF-MYH11A, PML-RARA (L, S and V), E2A-PBX1, TEL-AML1 and SIL-TAL1 fusion genes were tested in 23 cases and the results were negative. The JAK2V617F mutation was tested in five cases and one case was positive (Table IV).

## Discussion

In 2001, WHO defined MDS/MPN as a separate disease, however its laboratory characteristics have not been reported as a whole. In the present study, blood, bone marrow, immunological and genetic results of 53 MDS/MPN cases were analyzed and compared, demonstrating that the main features of MDS/MPNs are increased leukocytes, moderate anemia, the appearance of blasts and immature cells in the peripheral blood, an increased ratio of monocytes and at least one series of bone marrow dysplasia. Cytogenetic +8 and -7/7q- were more easily viewed. Tests for Ph- and AML-related chromosomal abnormalities were negative. Tests for BCR/ABL and the leukemia-related fusion gene were negative. The JAK2V617 gene mutation was observed. There were differences in the laboratory features of the different subtypes.

Compared with the WHO diagnostic criteria for CMML, the peripheral blood mononuclear cells in CMML were significantly increased (26.5%, 3.62×10^9^/l) and higher than the diagnostic criteria (≥10%, the absolute value of ≥1×10^9^/l), which is slightly lower than that identified by Wu *et al* (4.6×10^9^/l) (7). The ratio of mononuclear cells (26.5%) is similar to the study by Wang *et al*(8), who reported a lower absolute value of monocytes (5.6×10^9^/l). The most common feature in bone marrow was granulocyte dysplasia. Monocytic dysplasia was observed in 41.7% of CMML cases and this had not been identified in previous studies. Monocytic dysplasia included the appearance of primitive and immature monocytes, with mononuclear cells varying in size, nuclear lobulation, abnormal cytoplasmic granules and changes similar to the characteristics of plasma cells. The main feature of monocytic dysplasia which occured in CMML was granulocytic and monocytic proliferation, with granulocytic and monocytic dysplasia in bone marrow. Mononuclear cell infiltration (for example skin rash) is easily viewed clinically, which is different to the other three subtypes.

Differentiating CMML from secondary mononucleosis depends on a diagnosis via exclusion. The monocytes in CMML may undergo pathological changes. FCM analysis shows that the monocytes are CD56^+^ or CD2^+^, whereas CD13, CD15 and HLA-DR expression were reduced.

The leukocyte increase observed in aCML patients (23.9×10^9^/l) was higher than that reported by Gong *et al* (15.9×10^9^/l) (2) and lower than that reported by Onida *et al* (36×10^9^/l) (9). The main features of CMML are myeloid proliferation, higher proportion of naïve granulocytes in the peripheral blood (compared with other types of MDS/MPNs) and more easily observed myeloid dysplasia in bone marrow; this differs from the other subtypes. The eosinophil and basophil counts in the peripheral blood and bone marrow of CMML patients did not increase. The Ph chromosome and BCR/ABL fusion gene tested negative, which may differentiate CMML from CML.

A previous study (10) showed that the age of onset for JMML patients is <3 years old. In this study, the median age of onset in JMML patients was 3.8 years old. These results are similar, however slightly higher, than the 14 month age of onset reported by Lu (11). JMML patients in this study were more likely to have splenomegaly, fever and enlargement of lymph nodes, a high peripheral WBC count (32.7×10^9^/l), pathological monocytic changes, anemia and thrombocytopenia, and these are more severe than in the other groups. It is necessary to distinguish JMML from infection, Langerhans cell histiocytosis and idiopathic thrombocytopenic purpura.

In MDS/MPN-U patients, the WBC count was 7.5×10^9^/l, the Hb level was 100 g/l and the mean PLT count was 653.1×10^9^/l, These results differ from those which were reported in China (WBC, 9.9×10^9^/l; Hb, 85.8 g/l; and PLT, 518.5×10^12^/l) (3); this may be due to a fewer number of cases being analyzed. The first case in the MDS/MPN-U group exhibited anemia, leukopenia and significantly increased platelet levels of 1,112×10^12^/l. The bone marrow exhibited erythroid dysplasia, megakaryocyte proliferation and thrombocytosis.

The second case exhibited increased ring sideroblasts, immature red blood cells and immature myeloid cells in the peripheral blood, dry tap bone marrow, fibroplasia, splenomegaly and was also diagnosed with bone marrow fibrosis with increased ring sideroblasts. The third case presented with ring sideroblastic anemia with thrombocytosis and was diagnosed as MDS/MPN-U, RARS-T. The fourth case had significantly increased amounts of red blood cells and hemoglobin (185 g/l), whereas megakaryocytes developed dysplasia, causing thrombocytopenia. The second and fourth cases of MDS/MPN-U are not within the current MDS/MPN-U diagnostic criteria, however they exhibit characteristics of MDS and MPN. Bone marrow fibrosis with ring sideroblasts should not be diagnosed as MDS/MPN-U due to the insufficient proliferation of bone marrow fibroblasts. However, bone marrow fibrosis has been included as a class of MPNs, therefore, bone marrow fibrosis with ring sideroblasts should be classified as an MDS/MPN. These may require more laboratory data for confirmation.

Overall, 41.4% of the MDS/MPN cases had chromosomal abnormalities. More specifically, 40% of the CMML, 50% of the aCML, 33.3% of the JMML and 50% of the MDS/MPN-U cases had chromosomal abnormalities. The incidence of abnormal karyotypes was similar to those described in the international literature [36% erythropoiesis in CMML (9), 56–82% erythropoiesis in aCML (12) and 36–43% erythropoiesis in MDS/MPN-U (13,14)] and slightly lower than those described in the domestic literature [54.5% in aCML (2), 50% in JMML (15,16) and 62.5% in MDS/MPN-U (3)]. The probability of +8 and -7/7q^-^ was higher in the present study. The cytogenetic analysis of 23 cases showed no BCR/ABL1 or reproducible chromosomal abnormalities, as proposed by WHO. If patients have acute myeloid leukemia (AML) associated with specific chromosomal abnormalities, even if the primitive cells are <20%, it should be classified as AML.

Diagnosing MDS/MPNs currently relies on clinical manifestations, blood and bone marrow changes, no characteristic chromosome changes, and should, except BCR/ABL1, PDGFRα, PDGFRβ, PGFR1 and MLL genes. Detecting mutations in JAK2V617 and RAS mutation is valuable in the diagnosis of MDS/MPNs. The JAK2 gene has been identified as being positive in <20% of MDS/MPN patients (17); however, in this study, only one case was positive for the JAK2 gene among the five MDS/MPN cases. Thus, the incidence of JAK2 gene mutations in MDS/MPNs requires further study and exploration. Previous studies (18,19) showed that mutations in CBL, JAK2, MPL, NRAS, KRAS, RUNX1 and TET2 regions occur in MDS/MPNs; this may reveal the complex molecular characteristics of MDS/MPNs and give further indication of the different biological characteristics and prognosis of MDS/MPNs.

## Figures and Tables

**Table I:** Clinical symptoms of patients with MDS/MPNs.

Type of neoplasm	Age (years), median (range)	Gender (M/F)	Pallor weakness, n (%)	Fever, n (%)	Bleeding, n (%)	Hepatomegaly, n (%)	Splenomegaly, n (%)	Lymph node enlargement, n (%)	Ostealgia, skin rash, n (%)
CMML (n=24)	57 (33–71)	17/7	18 (75.0)	3 (12.5)	2 (8.3)	3 (12.5)	14 (58.3)	3 (12.5)	10 (41.7)
aCML (n=13)	68 (16–84)	6/7	13 (100)	6 (46.2)	2 (15.4)	2 (15.4)	7 (53.8)	0 (0)	1 (7.7)
JMML (n=12)	3.8 (14 days-16)	8/4	12 (100)	9 (75.0)	3 (25.0)	3 (25.0)	10 (83.3)	9 (75.0)	5 (41.7)
MDS/MPN-U (n=4)	68 (57–82)	2/2	3 (75.0)	3 (75.0)	2 (50.0)	2 (50.0)	2 (50.0)	1 (25.0)	4 (100)
Total (n=53)	58 (14 days-84)	33/20	46 (86.8)	21 (39.6)	9 (17.0)	10 (18.9)	33 (62.3)	13 (24.5)	20 (37.7)

M, male; F, female; CMML, chronic myelomonocytic leukemia; aCML, atypical chronic myeloid leukemia, BCR-ABL1^−^; JMML, juvenile myelomonocytic leukemia; MDS/MPN-U, myelodysplastic/myeloproliferative neoplasm, unclassifiable.

**Table II:** Main hematological features of patients with MDS/MPNs (mean ± SD).

Type of neoplasm	Hb (g/l)	WBCs (×10^9^/l)	PLTs (×10^9^/l)	Blast percentage	Immature myeloidcytes	Monocyte percentage	Monocytes (×10^9^/l)	Eosinophils (×10^9^/l)	Basophils (×10^9^/l)	BM blast percentage
CMML (n=24)	90.6±18.2	13.7±5.5	220.1±124.1	0.032±0.024	0.041±0.027	0.265±0.059	3.62±0.76	0.26±0.17	0.07±0.05	0.066±0.038
aCML (n=13)	79.4 ±11.7	23.9±10.4	112.5±52.3	0.033±0.023	0.105±0.039	0.020±0.007	0.60±0.06	0.24±0.13	0.25±0.09	0.024±0.013
JMML (n=12)	73.0 ±5.8	32.7±9.8	75.3±29.8	0.015±0.005	0.055±0.038	0.130±0.023	4.25±0.76	0.49±0.26	0.10±0.07	0.025±0.014
MDS/MPN-U (n=4)	100.0±18.6	7.5±3.96	653.1±210.5	0.010±0.007	0.045±0.025	0.060±0.011	0.45±0.02	0.20±0.13	0.07±0.02	0.017±0.015
Total (n=53)	83.1±24.6	19.8±8.1	158.7±108.2	0.024±0.015	0.058±0.031	0.152±0.034	3.00±0.51	0.30±0.17	0.07±0.02	0.024±0.021

Hb, hemoglobin; WBC, white blood cell; PLT, platelet; BM, bone marrow; CMML, chronic myelomonocytic leukemia; aCML, atypical chronic myeloid leukemia, BCR-ABL1^−^; JMML, juvenile myelomonocytic leukemia; MDS/MPN-U, myelodysplastic/myeloproliferative neoplasm, unclassifiable.

**Table III:** FCM abnormalities in blasts and myoloidcytes in MDS/MPNs.

	Blasts			FCM abnormalities in myeloidcytes							FCM abnormalities in monocytes					
			
Type of neoplasm	≥3%	CD2/5/7/56	CD117/45^a^	SSC decrease^b^	CD14/64^c^	CD10	CD33/15^c^	CD117/34	CD11b/13^d^	CD56	CD14/64^c^	DR	CD13/11b^d^	CD15	CD56	CD2/5/7^c^
CMML (n=7)	3	3	4	4	5	4	4	1	4	2	0	2	5	2	4	2
aCML (n=4)	2	1	3	2	3	3	3	1	3	0	0	0	3	0	1	0
JMML (n=3)	1	1	1	2	2	2	3	0	1	0	2	2	3	1	2	2
MDS/MPN-U (n=1)	0	1	0	1	1	1	1	1	0	0	1	0	1	0	0	0
Total (n=15)	6	6	8	9	11	10	11	3	8	2	3	4	12	3	7	4

FCM, flow cytometry; SCC, side scatter; HLA-DR, an MHC class II cell surface receptor encoded by the human leukocyte antigen complex on chromosome 6 region 6p21.31; CMML, chronic myelomonocytic leukemia; aCML, atypical chronic myeloid leukemia, BCR-ABL1^−^; JMML, juvenile myelomonocytic leukemia; MDS/MPN-U, myelodysplastic/myeloproliferative neoplasm, unclassifiable.

a Abnormal expression of CD117, CD45: increased CD117 and/or decreased CD45 (the change of mean fluorescene intensity ≥1/3 of a decade on log scale).

b Decreased SSC: The lower SSC limit of the maturing myeloid population decreased by ≥ 100 mean fluorescence channel (MFC; usually reached or was below the third linear scale) or the MFC of the whole population drifted downward by >150 MFC.

c Increased or decreased myeloid antigen (CD33, CD13, CD11b, CD15, CD64, CD10, CD14, HLA-DR) intensity scale compared with normal controls a log of a decade at least 1/3 of a decade on a log scale compared with normal controls; or at least 10% population express lymphocytic antigens.

d Lack of maturational spectrum or asynchronous expression of two myeloid-associated antigens in a population of interest (CD64/CD10, CD33/CD15 and CD11b/CD16/CD13 maturation patterns).

**Table IV:** Dysplasia, FCM and cytogenetic analysis in patients with MDS/MPNs.

Type of neoplasm	Erythroid dysplasia, n (%)	Myeloidic dysplasia, n (%)	Monocytic dysplasia, n (%)	Megakaryocytic dysplasia, n (%)	FCM (n=15)			Karyotype (n=29)		
					POS	INT	NEG	Normal	Abnormal	Abnormal karyotypes
CMML (n=24)	10 (41.7)	11 (45.8)	10 (41.7)	8 (33.3)	6/7	1/7	0	6/10	4/10	+8,-7,add(12)p(12),7q-
aCML (n=13)	5 (38.5)	11 (84.6)	1 (7.7)	7 (53.8)	4/4	0	0	3/6	3/6	+8, del(11)(q12), del(12)(q13)
JMML (n=12)	5 (41.7)	10 (83.3)	12 (100)	7 (58.3)	2/3	1/3	0	6/9	3/9	-7 (2 cases), -5
MDS/MPN-U (n=4)	3 (75.0)	4 (100)	1 (25.0)	3 (75.0)	1/1	0	0	2/4	2/4	+8, [+1, der(1)t(1;7)(q10;p10)]
Total (n=53)	23 (43.4)	36 (67.9)	24 (45.3)	24 (45.2)	13/15	2/15	0	17/29	12/29	+8 (3 cases), 7-involved-abnormal (5 cases), others (4 cases)

FCM, flow cytometry; POS, positive; INT, intermediate; NEG, negative; CMML, chronic myelomonocytic leukemia; aCML, atypical chronic myeloid leukemia, BCR-ABL1^−^; JMML, juvenile myelomonocytic leukemia; MDS/MPN-U, myelodysplastic/myeloproliferative neoplasm, unclassifiable.
